# Aeroparticles, Composition, and Lung Diseases

**DOI:** 10.3389/fimmu.2016.00003

**Published:** 2016-01-20

**Authors:** Carlos I. Falcon-Rodriguez, Alvaro R. Osornio-Vargas, Isabel Sada-Ovalle, Patricia Segura-Medina

**Affiliations:** ^1^Posgrado en Ciencias Biológicas, Universidad Nacional Autónoma de México, Mexico City, Mexico; ^2^Departamento de Investigación en Hiperreactividad Bronquial, Instituto Nacional de Enfermedades Respiratorias, Mexico City, Mexico; ^3^Department of Pediatrics, University of Alberta, Edmonton, AB, Canada; ^4^Laboratorio de Inmunologia Integrativa, Instituto Nacional de Enfermedades Respiratorias, Mexico City, Mexico

**Keywords:** air pollution, particulate matter, COPD, asthma, fibrosis, inflammation

## Abstract

Urban air pollution is a serious worldwide problem due to its impact on human health. In the past 60 years, growing evidence established a correlation between exposure to air pollutants and the developing of severe respiratory diseases. Recently particulate matter (PM) is drawing more public attention to various aspects including historical backgrounds, physicochemical characteristics, and its pathological role. Therefore, this review is focused on these aspects. The most famous air pollution disaster happened in London on December 1952; it has been calculated that more than 4,000 deaths occurred during this event. Air pollution is a complex mix of gases and particles. Gaseous pollutants disseminate deeply into the alveoli, allowing its diffusion through the blood–air barrier to several organs. Meanwhile, PM is a mix of solid or liquid particles suspended in the air. PM is deposited at different levels of the respiratory tract, depending on its size: coarse particles (PM_10_) in upper airways and fine particles (PM_2.5_) can be accumulated in the lung parenchyma, inducing several respiratory diseases. Additionally to size, the composition of PM has been associated with different toxicological outcomes on clinical and epidemiological, as well as *in vivo* and *in vitro* animal and human studies. PM can be constituted by organic, inorganic, and biological compounds. All these compounds are capable of modifying several biological activities, including alterations in cytokine production, coagulation factors balance, pulmonary function, respiratory symptoms, and cardiac function. It can also generate different modifications during its passage through the airways, like inflammatory cells recruitment, with the release of cytokines and reactive oxygen species (ROS). These inflammatory mediators can activate different pathways, such as MAP kinases, NF-κB, and Stat-1, or induce DNA adducts. All these alterations can mediate obstructive or restrictive respiratory diseases like asthma, COPD, pulmonary fibrosis, and even cancer. In 2013, outdoor air pollution was classified as Group 1 by IARC based on all research studies data about air pollution effects. Therefore, it is important to understand how PM composition can generate several pulmonary pathologies.

## Introduction

Urban air pollution is a serious problem around the world. Since the Industrial Revolution, the growing use of fuels, electricity demand, and mining activities have been the primary drivers of atmospheric pollution. It was not until after historic high air pollution events that scientists began the study of its impact on health. Three well-documented air pollution episodes occurred in the twentieth century: “The great air pollution disasters” or “The historic pollution episodes.” The first occurred in the Meuse river valley in eastern Belgium. The valley hosted a massive industrial zone with a diverse set of air pollution sources. On December 1930, a combination of low temperature, fog, and low wind speed resulted in the lack of air dispersion, and the consequence was seen as a large accumulation of gaseous and particulate air pollutants in the valley. In 2 days, 6,000 cases of unexpected deaths were observed, mainly impacting the elderly and individuals with preexisting heart and lung diseases (1). The second incident occurred on October 1948, in Donora, PA, USA. The heavily industrialized Monongahela River valley used soft coal as the main fuel. The episode began with persistent cool air and heavy fog and had the sharply irritating pungent odor of sulfur dioxide. While 1 to 2 deaths were expected during the time of the event, an astonishing 18–20 excess in deaths was attributed to the episode (1).

The third episode represented the most severe air pollution disaster and occurred in London during 4 days in December 1952. In the Thames Valley, the meteorological conditions were unusually intense, with cold, stagnant air, dense fog, and a rapid buildup of soot-filled air as a result of a thermal inversion. Approximately 4,000 deaths occurred during that period (2). The cause of death included pneumonia, bronchitis, and heart diseases. Prior to the episode, particle levels averaged a substantial equivalent of 500 μg/m^3^ of air, and sulfur dioxide levels averaged 0.15 ppm. During the episode, accumulated particles levels arose to 4,500 μg/m^3^, and sulfur dioxide level reached a substantial 1.3 ppm. The British Smoke Shade methods were used to estimate particles levels based on the dark color of the filter sampler (1). After three events, the scientists started studying the different pathologies or damage caused by air pollution (1). The preexistence of cardiopulmonary disease in individuals aged ≥45 years and during infancy, it was an important condition in 80% of the deaths (1).

In general terms, air pollution is made up of gases and particulate matter (PM). Especially, PM can be transported into the alveoli, depending on its size (3). In recent years, some researchers have reported that air pollution can produce cancer. Lately, outdoor air pollution was classified by the IARC as a group I carcinogen or proven carcinogen for humans (4). However, exposure to PM also produces several other diseases in the respiratory system. Therefore, it is important to establish how the composition of PM can induce several pulmonary pathologies.

## Particulate Matter

Particulate matter is defined as solid and/or liquid suspended in the atmosphere, also named aerosol (5). It is generated chiefly through two processes, natural and anthropogenic. The natural process includes phenomena that take place on the earth, such as, sea sprays (6), volcanic eruptions, spontaneous forest fires, and soil erosion (7). The second process involves emissions to the atmosphere, mainly from traffic, other forms of transportation, and industrial sources, such as electricity generation, mining, welding, and building (8). In general, any form of fuel burning, for instance, wood, gas (9), oil-derived diesel, and gasoline (10), generates PM.

Many different types of particles can be found in the atmosphere. If particles are emitted directly to the atmosphere, they will be named primary PM (11, 12), but if they are formed in the atmosphere by gas-to-particle conversion processes, they will be named secondary particles (6). Mineral dust, metals, soot, salt particles, pollen, and spores constitute primary aerosols. On the other hand, secondary aerosols are formed by gases such as sulfates, nitrates, and organic compounds (5). These processes follow three steps that can increase particle size or modify its composition. Nucleation-mode is the first step in new particles generation (13) and depends on gases concentration, humidity, and temperature in the atmosphere (11), and transition of the gaseous phase to liquid or solid phase by condensation or chemical reaction, forming the first nuclei or particles in the atmosphere (5).

**Figure 1:**
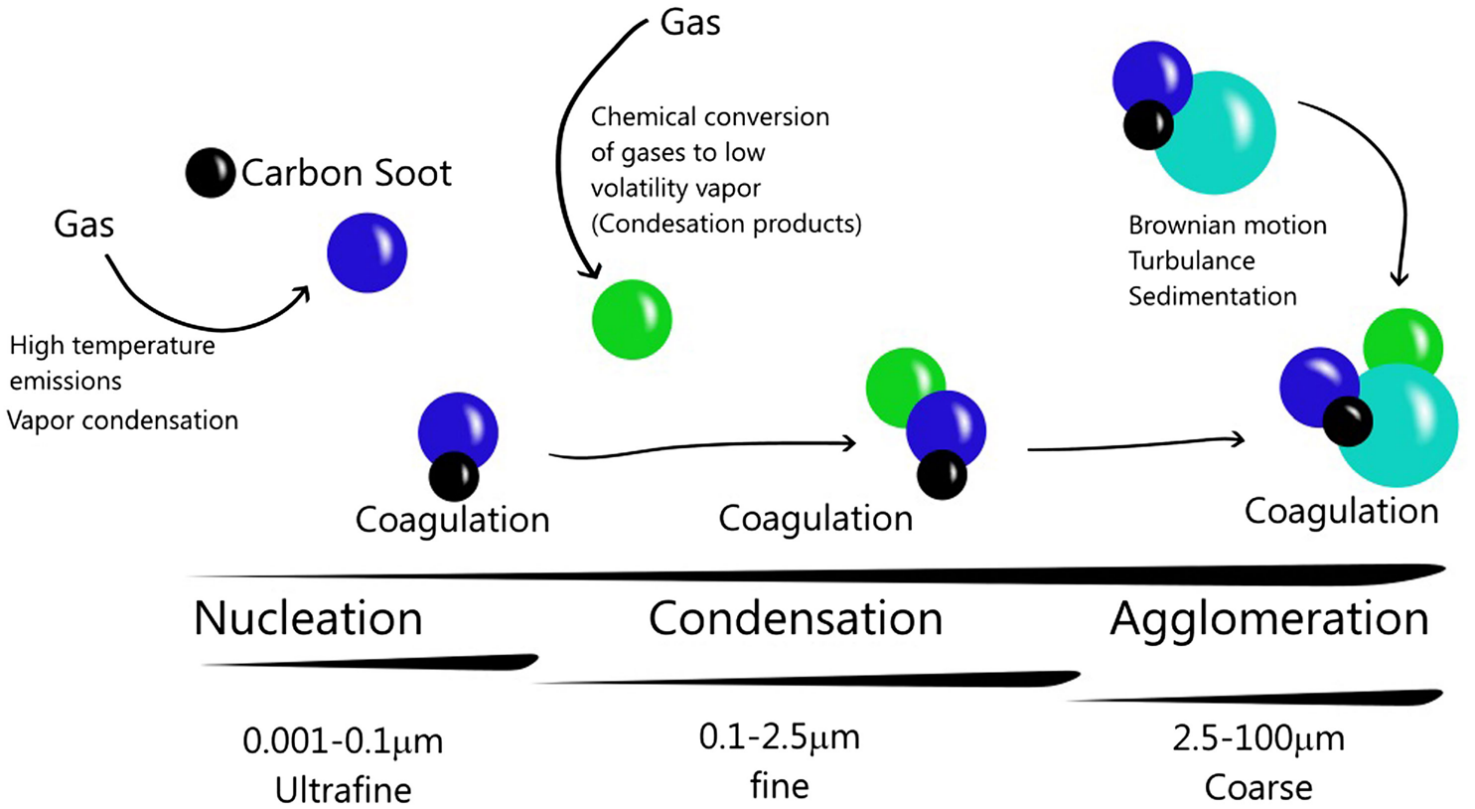
**Particulate matter and its atmospheric dynamics**. Particles nucleation is generated by gases emission. Condensation can occur by cooling, producing particles. The interaction between primary particles and secondary particles constitute the coagulation. In this way, the particles can increase their size and composition.

The second step is a condensation of hot gases, originating primary aerosols. This event is similar to nucleation (11). The final step in the aerosol formation is coagulation. Whole aerosols formed in previous steps can begin to agglomerate by Brownian motion (14) or turbulence and contact between particles (1). Consequently, particles grow in aerodynamic size (15) forming secondary particles from primary particles (Figure 1).

## Composition and Size

There are different types of PM depending on its source and its composition, e.g., Diesel exhausts particles (DEP), residual oil fly ash (ROFA), and Utah Valley urban air particles (UAP). The first one is produced by diesel combustion and is constituted by transitional metals, such as vanadium and zinc (16), and also by Polycyclic Aromatic Hydrocarbons (PAHs). ROFA is a complex mixture of sulfates and nitrogen compounds, carbon and metals (primarily vanadium) (17). UAP were identified in the United State of America, but became a general denomination for any UAP around the world, due to its high transitional metal levels (18).

**Figure 2:**
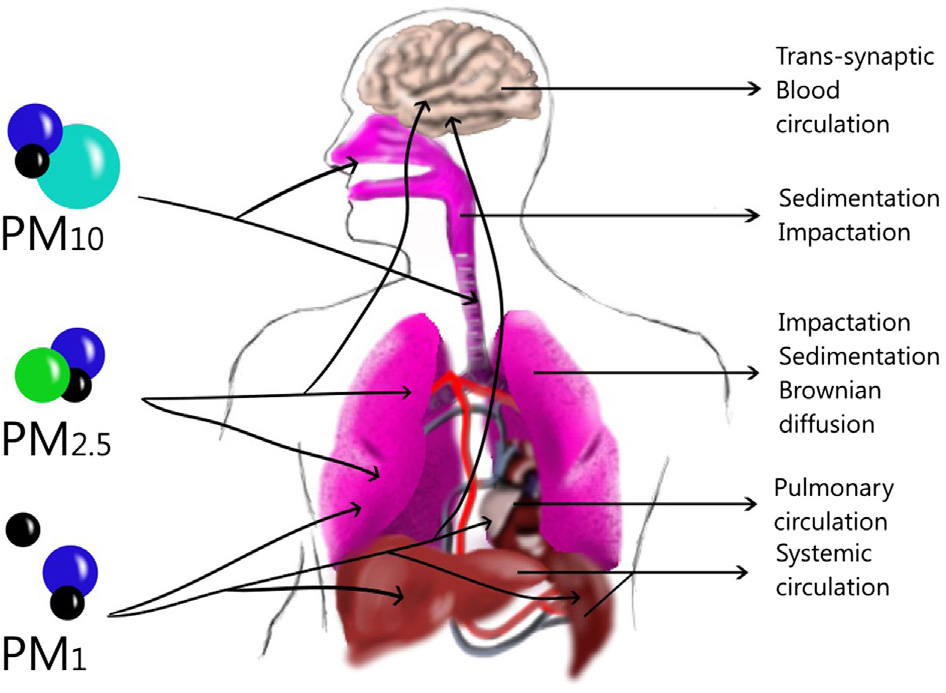
**Size and Dynamic of particles in the lung and other tissues**. Large particles can be deposited in upper airways through sedimentation or impaction while in the lower airways Brownian diffusion can deposit them in the alveoli. Ultrafine particles can translocate to blood-circulating and be deposited in the liver, spleen or brain, although they might also penetrate through trans-synaptic mechanisms.

Particulate matter is commonly formed by different compounds (Table 1). PM composition can be different among cities, depending on the predominant emission sources. Another important characteristic of particles is the size that depends on the emission sources such as primary aerosol and atmospheric dynamic as a secondary aerosol, already described. The size plays an important role in human airways because it will define the deposition site in the lung. The deposition of aerosol in the human lung occurs through a combination of inertial impaction, gravitational sedimentation, and Brownian diffusion (19). In the atmosphere, different PM sizes can be found, such as the coarse fraction (PM_10–2.5_) that can penetrate into the upper airways (20) and is deposited through an impaction or sedimentation process (19). The fine fraction (PM_2.5_) is deposited in the lung, especially in the alveoli (20) through sedimentation and Brownian diffusion processes (19), although it could pass to the systemic circulation (20). PM_1_ is deposited mainly by Brownian diffusion in the lung (19), but these particles can be translocated from sites in the lung through systemic circulation (21) to the liver, spleen, heart (22), or brain (23). However, they can also arrive to the brain through the olfactory bulb by a trans-synapsis mechanism (23) (Figure 2).

**Table 1:** **The composition of particles**.

Composition	Elements	Reference
Metals	K, Ca, Ga, Pb, Sr, Zr	(16, 24–26)
	Ba, Na, Li, Be, Ti, Sn, Mg	
	Al, Cs, Bi	
	In	
	Sb	
Transitional metals	Cr, Mn, Fe, Ni, Cu, Zn	(16, 24, 25, 27)
	Cd, Au, V, Hg, Nb, Tl, Co	
	Mo	
	Zr	
	Rb, Ag	
Non-metals	B, As, Se	(24, 25)
	S	
	Sb	
Lanthanides and actinides	Sm, U	(24, 25, 27)
	Tb	
	Ce, La	
Biologicals	Glucans	(20, 28)
	Endotoxins	
	Pollens	
	Viruses	
Carbon	Elemental	(29)
	Organic	
PAHs	(AcPy) acenaphtylene	(27)
	(Ant) anthracene	
	(BaA) benzo[a]anthracene	
	(BaFL) benzo[b] fluoranthene	
	(BkFL) benzo[k] fluoranthene	
	(BaP) benzo[a]pyrene	
	(Bg,h,iP) benzo[ghi]perylene	
	(BaP-TEQs) Benzo[a]Pyrene-Toxic	
	(Chr) chrysene	
	(Flu) fluorine	
	(Fl) fluoranthene	
	(Nap) naphthalene	
	(InP) indeno[cd] pyrene	
	(BkF) dibenzo[a,h]anthracene	
	(Phe) phenanthrene	
	Pyrene	
Others	Ammonium sulfates and nitrates	(7, 18, 30)
	Paraformaldehyde	

## Specific Composition and Damage

The complex mix of PM can produce different changes in the tissues, depending on its composition, which includes a water-soluble or a water-insoluble fraction (31). The water-soluble fraction can produce cell signaling, expression of inflammatory mediators, oxidative stress (32) that generates DNA damage via a transition metal-dependent OH formation, implicating an important role of H_2_O_2_ (33). *In vitro* experiments in BEAS-2B have demonstrated that oxidant generation and the concentration of inflammatory cytokine were higher in an exposure with the water-soluble fraction than in an exposure with the insoluble fraction; the production of IL-8 by the former cells increased as well. Likewise, *in vivo* intratracheal instillation of both water-soluble and insoluble fractions in rats also increased neutrophil incursion and lavage protein concentrations. However, both neutrophil and protein elevation were greater after the exposure to water-soluble fraction (32). Furthermore, water-soluble and insoluble organic aerosols substantially contribute to the oxidative properties of ambient PM (34).

Some researchers have applied statistical methods to demonstrate that certain elements of the particles can produce specific changes. They have used a multivariate technique that analyzes the inter-correlated quantitative dependent variables, called the Principal Component Analysis (PCA) (35). This analysis has demonstrated a strong relationship between IL-6/TNF-α secretion with the presence of Cu and Zn from anthropogenic sources in Mexicali (36). Another study, using BEAS-2B cells exposed to France’s PM from a different season (2008/2009) indicated that inorganic elements and ions were rather related to early oxidative events, whereas PAHs were rather related to later oxidative damage and cytokine secretion such as IL-8 (27). The exposure also produced ROS after PAHs metabolism through the cytochrome P450 activation (37). Some chemical components are preferentially associated with these early oxidative events. Other metals and PAHs have also been associated to oxidative damage and/or cytokine secretion (27).

## Respiratory Effects

Particulate matter is easily deposited on bifurcations or angle ramifications of the bronchial tree due to air flow and turbulence, increasing PM interaction with the mucous membrane through an impact process (38). Once deposited on a particular region in the lung, it can penetrate or be absorbed by the mucous layer, generating local damage (39). Many researchers have done *in vitro* experiments with DEP or carbon black. They have analyzed the effects of particle accumulation in macrophages and their phagocytic capacity (40). PM can produce damage to the whole respiratory apparatus, increasing cellular permeability and reducing the mucocilliary activity by ROS production and cytokine releases. Since 1980, many reports have mentioned that exposure to PM increases cancer and deaths. It is well known that exposure to PM cause pulmonary diseases such as COPD, asthma, and fibrosis (41).

## Physiological Alterations

Pulmonary function assessment is the main non-invasive procedure to evaluate respiratory health, identifying ventilation alterations, such as restrictive or obstructive pathologies. For example, FEV_1_ assesses the volume of forced exhalation in the first second. Another parameter is the forced vital capacity (FVC) that evaluates the amount of air exhaled during the FEV assay. Jie et al. (42) evaluated FVC and FEV on individuals during cooking with coal and non-coal fuels, as well as PM indoor concentration in the kitchen and living room. Despite the absence of outdoor particles, their results showed a significant increase in the relative concentration of PM_2.5_ in the indoor. The coal smoke was associated with 31.7% decrease in FVC, and 42.0% decrease in FEV_1_. They conclude that in the kitchen, the fine particles’ relative concentration produces a significant effect on the FVC and FEV_1_. On the other hand, children exposure to PM_10_ causes the loss of 23 ml in the FEV_1_ test for every 1 μg/m^3^ increment of PM_10_ in the atmosphere (43). The same finding was observed in women; a reduction of 5.1% in FEV_1_ per each 7 μg/m^3^ increment of PM_10_ (44) was found. In this regard, Forbes et al. (45) mentioned that FEV_1_ is associated with increased outdoor PM_10_ concentration_._ In their analysis, they mentioned that increases in 3 μg/m^3^ PM_10_ was associated with a loss of 28 ml in FEV_1_, mainly in men, elderly people, and ex-smokers.

Also, a Swiss study (SAPALDIA) aimed to evaluate respiratory health in the adult population and the potential association between long-term exposure to air pollution and respiratory health, concluded that PM_10_ reduced 3.4% the FVC per each increment of 10 μg/m^3^ of PM_10_ (46). Even more, patients with a diagnosis of COPD or asthma had a major reduction of the FEV_1_ when exposed to fine and ultrafine particles (47). Animal models have also been used to evaluate physiological parameters after PM exposures. Barometric plethysmography can also measure respiratory frequency and tidal volume. Exposure to PM increases both parameters (66–103 bpm and 97–190 ml, respectively) in rats (48). Another parameter, Penh (enhanced pause), has been used as well in plethysmographic studies (49). In the murine model, after intranasal instillation of PM1640 (standard reference material), the Penh index increased in a dose-response manner (50). The respiratory physiological changes could be generated by several mechanisms, like the release of molecular mediators that affect the cells, tissue, or systems after the PM exposure.

## Immune Response

Perivascular and peribronchiolar inflammation increases after DEP exposure (51). Titanium dioxide nanoparticles (TiO_2_) rise the number of neutrophils and macrophages recruited in the bronchoalveolar lavage fluid (BALF) (52). In healthy humans, the experimental exposure to DEP (300 μg/m^3^/h) increased the percentage of inflammatory cells (neutrophils), B and T lymphocytes, and mast cells in the lungs (17, 53). Intratracheal instillation (3.3 mg/kg) of Mexico City particles (PM_2.5_ and PM_10_) in a rat model increased the number of inflammatory cells in the lungs (54). This concentration induced lymphocytosis, but higher concentration, such as 5 mg/m^3^ leads to lymphopenia (20). Some inflammatory proteins, such as IL-1 (55), IL-17 (56), IL-6, IL-8, and TNF-α, increase (57). Exposure to DEP or UAP also increased the same cytokines (58) in *in vitro* experimental models (59). Also, other cytokines as MIP-1 (2), GM-CSF, IL-1, 2, 4, 5, 10 (60), and IL-13 (17), are affected. Especially, exposure to DEP increases IL-4 (17) and IgE (61). This inflammation process permits diseases development through cytokines activation.

## Oxidative Stress

Exposure to fine or ultrafine particles induces ROS-mediated oxidative stress, altering cellular permeability in epithelial cells (62) due to their organic or inorganic content (63). A primary form of ROS is the hydroxyl radical formed by hydrogen peroxide after exposure to PM (64). Also, PM_2.5_ can produce superoxide leading to the formation of hydrogen peroxide (65). H_2_O_2_ is a main free radical in the lung; it can produce cell damage by oxidant stress. Alveolar macrophages and epithelial cells generate oxidants (18). Exposure to Mexico City’s PM changed mRNA expression of several markers of oxidative stress in an *in vivo* model. Especially, PM_10_ from the industrial and residential zone induced a significant 3.2-fold and 3.9-fold increase, respectively, as well as the stress-inducible protein HO-1. PM from Industrial area led to a significant 2.5-fold increase in the receptor for oxidized lipoproteins LOX-1 24 h after exposure (54). ROS function as intermediary signaling molecules and can activate the Tyrosine-kinase receptor, MAP kinase, NF-κB, and Stat-1 (66). These signaling pathways activate the transcription and gene expression of molecules related to inflammation, such as TNF-α and IL-1β (67), fibrosis, and apoptosis (18, 66). Oxidative stress can produce damage to DNA inside the lungs, as a consequence, cells die (62). In subjects with or without COPD or lung fibrosis, oxidative stress can regulate the NF-κB transcription, stimulating inflammatory cytokine synthesis (68, 69); exposure to PM can also increase exacerbations and the lung damage.

## Asthma

Asthma is a major health issue around the world, which is characterized by airway hyperresponsiveness (70), obstruction (71), and chronic inflammation (72). Another important aspect of this condition is the reversibility of airway obstruction, either spontaneously or following treatment (73). In the asthmatics patients, their pulmonary cells respond to an allergen, producing a Th2 response, including IL-4 and IL-13 (mucous metaplasia), which are required to initiate this response and drive allergen-specific IgE synthesis by B cells; IL-3, which drives basophil development. IL-3, IL-5, and GM-CSF can regulate eosinophil recruitment (74). Also, other interleukins such as IL-17 promote the neutrophilic reaction (75). All the proteins above maintain the asthmatic process in the airways.

Some reports found that the exposure to DEP activates Th2 response and leads to the production of IL-17A (75) and mucous in the bronchiolar epithelium. On the other hand, UAP exposure leads to high level of IgG (animal models) or IgE (humans) production (76) arising the asthma attack (77). Acute exposures to PM can activate the Th2 response, inhibiting INF-γ production (78) and promoting an asthmatic condition. Probably, the chronic exposure enhances Th1 response by activation of IL-12 (79), and in turn trigger to INF-γ (80). Th1 response strongly suppresses the Th2 response (79).

In the asthma animal model, it has been described that exposure to PM increases the asthmatic process, indicating that particles function as an adjuvant in the generation of *de novo* asthma in mice (76). This characteristic depends on the exposure site because the particles include different components that differ from site to site or city to city (57). PM could be similar to aluminum hydroxide, an indispensable adjuvant in vaccines or the asthma animal model. Both adjuvants, as well as PM, will produce oxidative stress by increasing the production of reactive species oxygen (ROS), IL-1, IL-8, and maturation of B lymphocytes (81). The second mechanism could be related to Th2 response in the lung and also to the production of inflammatory mediators such as IgE or IgG (82).

## Chronic Obstructive Pulmonary Disease

Chronic obstructive pulmonary disease (COPD) is a chronic progressive disease that is characterized by an airflow limitation (83), that can be observed in FEV_1_ or FVC and peak expiratory flow (PEF) (84). This obstruction is not reversible like in asthma. Exacerbation of COPD could be caused by bacteria, viruses (84), cigarette smoking (85), and exposure to indoor and outdoor air pollution (86). It is characterized by chronic inflammation of the airways and lung parenchyma, especially of neutrophils, activated macrophages, and lymphocytes (87). The patients present increased levels of IL-6, TNF-α, and IL-1β (88). A close correlation between high levels of air pollution and COPD clinical manifestation has been described. Patients with a diagnosis of COPD are more susceptible to urban particles, particularly elderly women and patients with severe COPD (89). Chen et al. (90) found a correlation between PM levels (PM_2.5_ and PM_10–2.5_) and increased COPD hospitalizations in Vancouver.

In patients with stable COPD showed an increase of proinflammatory mediators such as IL-6, IL-1β, TNF-α, and IL-8, produced by macrophages or epithelial cells (2). IL-6 increased in the systemic circulation in patients during exacerbations, whereas TNF-α and IL-1β was associated with a muscular mass decrease (88). A hypothetical response after PM_10_ exposure might be that particles produce NF-κB activation, and therefore, increase inflammation and exacerbations in patients with COPD (91). Transcription of TNF-α and IL-1β genes are known to be regulated by NF-κB (67).

## Pulmonary Fibrosis

Pulmonary fibrosis is a restrictive disease that presents an irreversible decrement of the vital capacity (63). Furthermore, some cells are implicated such as fibroblast, myofibroblast (92), and macrophage (93), which produce an excess of extracellular matrix components (94) and the pulmonary remodeling like an irreversible distortion of the lung’s architecture (95). In this event, the deposit of collagen fibers is stimulated by TGF-β (96), a potent mediator of fibrogenesis (97). Furthermore, this response is also involved in the production of IL-4 and IL-13 (98). Exposure to ambient particles could lead to pulmonary fibrosis (99), especially the exposure to elements or chemicals such as Al, Si, carbon black, TiO_2_, silicon oxide, talcum powder, asbestos, and other fibers can cause epithelial damage and rise the levels of IL-2 (100) and IL-8 (66).

PM_10_ collected in Mexico City can induce increases in the PDGF, a potent mitogen and chemotactic factor for interstitial cells (101), and together with NF-κB, are indispensable in survival factors that inhibits apoptosis and promotes proliferation (102); it also provokes myofibroblast differentiation (66) and production of collagen fibers in the lung (101). Another factor is the proteases activity (20) that increases in airway epithelial A549 cells exposed to PM_10_ of Mexico city. It was demonstrated an increase in protease activity, especially of MMP-2 and MMP-9 and a decrease in E-cadherin and β-catenin expression (103).

During 5 days, the exposure to TiO_2_ produced a small increase in procollagen in rat tracheal explants. After 7 days, fine TiO_2_ significantly increased PDGF-β, TGF-α, or TGF-β levels, compared with animals exposed to ultrafine TiO_2_; both particle samples produced similar increments of PDGF-A (104). These changes could be associated with oxidative stress, which generates inflammatory infiltrate (99). PM generate ROS, which produces a proinflammatory activity and cytotoxic effects. Proinflammatory effects are mediated by the MAP kinase and NF-κB cascades that are responsible for the expression of cytokines, chemokines, and adhesion molecules (105). In the bleomycin animal model, the exposure to carbon black nanoparticles increased neutrophils and macrophages in the lung. However, lymphocytes or eosinophils do not increase (106), and pulmonary fibrosis worsened in the mouse model (107). Intratracheal instillation of PM increases fibrosis (108) and its fibrogenic mediators such as KC, IL-6, CCL2, and TGF-β1 (109). Metals represents the major source of ROS in PM, which can activate mitogen proteins or nuclear regulating as; nuclear factors such as AP-1 and NFAT (110). However, other elements of PM also produce proteins activation by ROS, the exposures to particles worsen damage in patients with pulmonary fibrosis.

## Cancer

Recently, outdoor air pollution was classified as a group I carcinogen by the International Agency for Research on Cancer (4). Particles contain two major components that produce oxidant stress, PAHs, and metals. Both are strong mutagenic and carcinogenesis agents (111), and it has been associated with markers of genetic damage, which may increase the frequency of human cancer (112). Especially the cancers of the trachea, bronchus, or lung represented approximately 7% of total mortality attributable to PM_2.5_ in 2010 (113). Some studies showed that lung cancer among non-smokers can result by exposure to PM (114), however, the risk of developing lung cancer is higher in smokers (115).

Exposure to UAP or DEP can induce DNA single-strand break, producing a nucleoside, 8 hydroxyguanine, or 8-oxo-7,8-dihydro-2-o-desoxyguanosine (116), that is a predominant product of free radicals, which causes DNA adducts by oxidation (117). Formation of adducts is generated by PAHs metabolism of CYP1A1 and GSTM1 (116). Some reports have mentioned that *in vivo* exposure to PAHs increases the rate of chromosome aberration, and micronuclei in lymphocytes. In this regard, sister chromatide exchange has been found in lymphocytes of both policemen and drivers in China (112). Furthermore, there is evidence that PAHs can generate deletion (p) of an arm on chromosomes, as well as K-ras and P53 mutations (116), the principal oncogene and tumor suppressor, respectively.

## Conclusion

**Figure 3:**
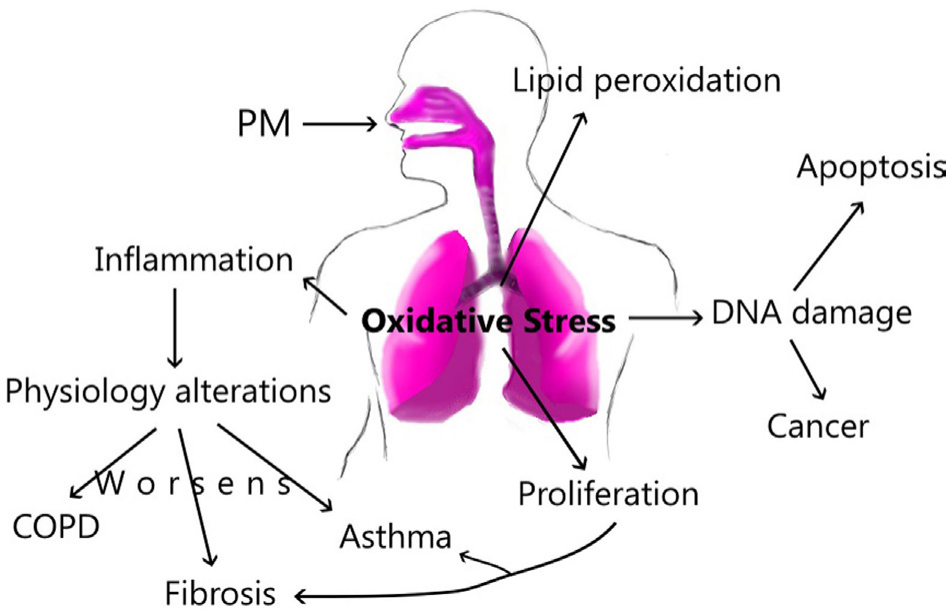
**The principal route of damage after PM exposure**.

Our ambient plays an important role in the composition and size of the particles; this dynamic process is the main factor to produce specific damage along the airways. In this way, the exposure can increase inflammatory factors and cellular recruitment in the lung, which promotes physiology alterations, resulting in pulmonary diseases as COPD and asthma. Likewise, PM can activate other cellular mediators that produce pulmonary fibrosis. Acute exposure to PM can activate Th2 immune responses; however, chronic exposure changes this profile by activation of Th1, and it triggers pro-fibrotic cytokines as well. The PM components are associated with different damage in accordance with elements such as water-soluble or insoluble fraction. However, all components presents in the PM, form a final complex mixture that will produce or activate inflammatory processes, damage or ROS in the lung. All this changes harm the epithelium, increasing epithelial permeability. In patients with pulmonary diseases, the exposure to PM increases the changes and the lung damage (Figure 3).

## Author Contributions

CF-R and PS-M: responsible for the general design and supervision of the review, writing of the manuscript, and final approval of the version to be published. AO-V and IS-O: writing of the manuscript and final approval of the version to be published.

## Conflict of Interest Statement

Authors have no conflict of interests of any kind to declare about this manuscript. The manuscript was produced or written in the absence of any commercial or financial relationships that could create a potential conflict of interest. The Review Editors Daisuke Kamimura and Hiroki Kimura and the Handling Editor, Masaaki Murakami, declare their shared affiliation, and the Handling Editor states that the review process nevertheless met the standards of a fair and objective review.
